# Reactive Oxygen Species-Mediated Tumor Microenvironment Transformation: The Mechanism of Radioresistant Gastric Cancer

**DOI:** 10.1155/2018/5801209

**Published:** 2018-03-27

**Authors:** Huifeng Gu, Tianhe Huang, Yicheng Shen, Yin Liu, Fuling Zhou, Yanxia Jin, Haseeb Sattar, Yongchang Wei

**Affiliations:** ^1^Department of Radiation and Medical Oncology, Zhongnan Hospital, Wuhan University, Wuhan, Hubei 430071, China; ^2^Department of Clinical Oncology, First Affiliated Hospital, Medical School of Xi'an Jiaotong University, Xi'an, Shanxi 710061, China; ^3^School of Medicine, University of Texas Medical Branch, Galveston, TX 77555, USA; ^4^Department of Hematology, Zhongnan Hospital, Wuhan University, Wuhan, Hubei 430071, China; ^5^Department of Clinical Pharmacy, Wuhan Union Hospital, Affiliated to Tongji Medical College, Huazhong University of Science and Technology, Wuhan, Hubei 430022, China

## Abstract

Radioresistance is one of the primary causes responsible for therapeutic failure and recurrence of cancer. It is well documented that reactive oxygen species (ROS) contribute to the initiation and development of gastric cancer (GC), and the levels of ROS are significantly increased in patients with GC accompanied with abnormal expressions of multiple inflammatory factors. It is also well documented that ROS can activate cancer cells and inflammatory cells, stimulating the release of a variety of inflammatory cytokines, which subsequently mediates the tumor microenvironment (TME) and promotes cancer stem cell (CSC) maintenance as well as renewal and epithelial-mesenchymal transition (EMT), ultimately resulting in radioresistance and recurrence of GC.

## 1. Introduction

Gastric cancer (GC) is the second most frequently diagnosed cancer and the second leading cause of cancer-related mortality in China [1]. Almost one million new cases are estimated to occur worldwide every year [2]. Radiotherapy (RT) can optimize outcomes in patients with gastric cancer [3]. However, the impact of RT is hindered by a frequent development of resistance to the treatment [4]. Radiotherapy causes tissue damage in two different ways, a direct damaging effect from radiotherapy itself and an indirect effect resulting from the alteration of cellular pathways [5]. Radiotherapy can generate DNA breaks and induce cell apoptosis to indirectly militate against the antitumor treatment by inducing the reactive oxygen species (ROS). ROS are products of an excessive oxidative phosphorylation in mitochondria, as well as products of peroxisome-mediated *β*-oxidation of branched and very long-chain fatty acids (VLCFAs) [6], which regulate a variety of important signaling pathways for cell proliferation and survival.

Chronic low-level increased ROS can activate the change of the tumor microenvironment. Radiotherapy typically causes chronic oxidative stress and induces higher levels of ROS. The haemal levels of ROS in gastric cancer patients are obviously increased, along with the abnormal expression of factors such as P38 which modulates the expression of inflammatory factors [7–9]. ROS also directly alter the tumor microenvironment by activating cancer cells and inflammatory cells, which in turn release a variety of inflammatory factors to promote CSC renewal [10], leading to therapeutic resistance [11].

### 1.1. Effects of Radiotherapy in Gastric Cancer

#### 1.1.1. ROS-Associated Radioresistance in Gastric Cancer

**Figure 1:**
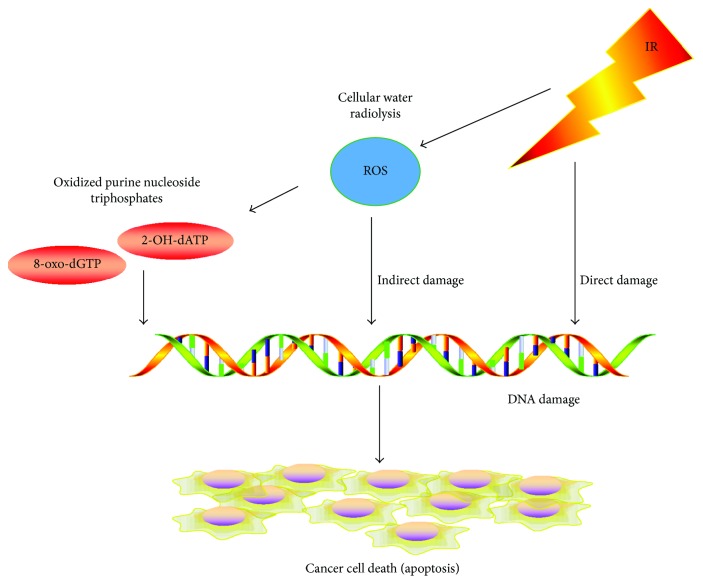
Radiotherapy and ROS promote antitumor effects. Radiotherapy irradiation causes cellular death through direct DNA breaks and indirect ROS effects. ROS induces 8-oxo-dGTP and 2-OH-dATP into genomic DNA, which leads to tumor cell apoptosis.

The biological effects of radiotherapy are mainly a consequence of DNA damage, such as breaks in the double-strand (ds) structure. These breaks can be directly caused by interactions between rays and DNA molecules, or indirectly from ROS-related cellular water radiolysis [12] (summarized in Figure 1). High levels of ROS suppress tumor growth through the inhibition of cell proliferation and induction of apoptosis and senescence. Incorporation of oxidized purine nucleoside triphosphates, such as 8-oxo-2′-deoxyguanosine triphosphate (8-oxo-dGTP) and 2-hydroxy-2′-deoxyadenosine triphosphate (2-OH-dATP), into genomic DNA plays an important role in apoptosis induced by ROS [13]. In addition, some studies confirm that tumor-infiltrating lymphocyte (TIL) can be attracted by ROS and exert their antitumor effects [14]. However, some cancer cells can survive ROS by activation of DNA repair and the antioxidant system [15]. Consequently, both activation of cellular DNA damage checkpoints and the ability to repair DNA in cells like CSCs contribute to cellular survival after receiving radiotherapy [10].

#### 1.1.2. ROS-Related Alterations in the Tumor Microenvironment after Radiotherapy

Radiotherapy can break the DNA of tumor cells and increase the levels of ROS, leading to damage in tumor cells and changes in the microenvironment. After radiotherapy exposure, normal and tumor tissues show inflammatory responses, including vascular trauma, tissue edema, and hypoxia. Pulmonary fibrosis is one of the most undesired side effects of RT. Studies have confirmed that some RT can cause acute lung injury, and the connective tissue growth factor (CTGF) mediates a chronic inflammatory response resulting in pulmonary fibrosis [16, 17]. Myofibroblast expansion and progressive deposition of the extracellular matrix can be observed in this process. The radiotherapy-induced vascular trauma, tissue self-healing, and immune cell infiltration usually cause an increased demand for oxygen, and the following hypoxic environment activates hypoxia-inducible factors (HIFs) [11]. The HIFs, particularly HIF-1*α* and HIF-2*α*, regulate tumor cell proliferation, migration, and angiogenesis by regulating glucose metabolism and ROS production [18, 19]. The hypoxia also influences the immune system by recruiting immune cells, such as tumor-associated macrophages (TAMs), T-cells, B-cells, and myeloid-derived suppressor cells (MDSCs) [20]. Whether HIFs function positively or negatively in the tumor immune response is not clearly understood. In addition, RT causes tumor cell death and inflammatory infiltration, which induce the release of tumor antigens and trigger antigen-presenting cells [21]. RT also promotes dendritic cell (DC) recruitment and a T-cell immune response through RT-induced IgM targeting of the necrotic tumor cells. The inflammatory environment within tumors can also attract TAMs and T-cells to suppress or promote tumor growth [22]. The microenvironment is deeply changed after RT in response to the effects of ROS, which results in the transformation of TME and contributes to resistant cancer cells.

### 1.2. ROS-Mediated Tumor Microenvironment Transformation in Gastric Cancer Patients

**Figure 2:**
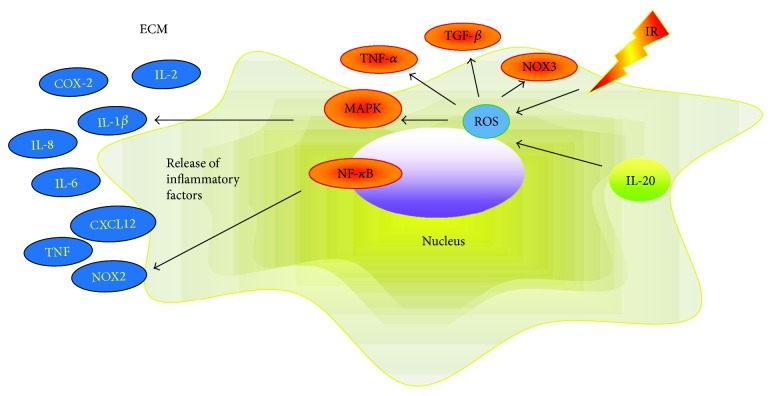
ROS mediate TME alterations. ROS can activate TNF-*α*, TGF-*β*, MAPK, NOX3, and NF-*κ*B signaling pathways and promote the release of inflammatory factors TNF, NOX2, IL-6, IL-2, IL-8, and CXCL12, leading to tumor microenvironment changes and the development of tumors.

Elevated levels of ROS are closely related to changes in the tumor microenvironment. The interaction between ROS and inflammation is an important pathogenic factor for GC carcinogenesis. Studies have shown that inflammatory mediators, such as cytokines and growth factors, can regulate nitrogen oxides (NOX) to produce ROS [23]. IL-20 stimulates ROS production through the activation of the signal transducer and activator of transcription 3 (STAT3), protein kinase B (AKT)/phospho-c-Jun NH(2)-terminal kinase (JNK)/extracellular signal-regulated kinase (ERK) signals [24]. As an effector molecule, ROS attract white blood cells involved in inflammation and tissue damage. Many studies have shown that ROS participate in carcinogenesis by activating inflammatory mediators, thus triggering an inflammatory microenvironment [25]. In Kupffer cells, ROS induce the release of inflammatory mediators by activating P38 to revitalize mitogen-activated protein kinase (MAPK) and nuclear factor-kappa B (NF-*κ*B) [26]. High levels of ROS can also activate tumor necrosis factor alpha (TNF-*α*), protein (p65), and transforming growth factor beta (TGF-*β*) and downregulate the inhibitor of kappa B alpha (I*κ*B*α*) to mediate the release of inflammatory mediators [23, 27, 28]. In addition, inflammatory cytokines can be released through the signal transducer and activator of transcription 1 (STAT1) signaling pathway, which is activated by ROS [29, 30]. ROS activate NF-*κ*B, TNF-*α*, and STAT3 signaling pathways in inflammatory cells and tumor cells to release TNF, NOX2, IL-6, IL-2, IL-8, and CXCL12 involved in the change of TME [23, 27] (summarized in Figure 2 and Table 1). Our former researches and other groups have confirmed that patients with GC are in a status of oxidative stress [32] accompanied by abnormal expression of a variety of inflammatory factors, including IL-1*β*, IL-6, and COX-2. Therefore, ROS may stimulate tumor cells to proliferate and resist apoptosis while promoting the development and progression of GC by impacting the tumor microenvironment.

### 1.3. Transformed Tumor Microenvironment Promotes Gastric Cancer Development and Radiotherapy Resistance

Radioresistant GC cells have stem cell-like features. Several studies have shown that cancer stem cells (CSCs) play an important role in developing resistance and recurrence of cancer. The change of the tumor microenvironment after radiotherapy can activate CSC renewal and epithelial-mesenchymal transition (EMT) [33, 34]. CSCs display an EMT phenotype that is resistant to conventional therapies. Self-renewal of such cells is the main cause for treatment resistance and recurrence of GC (summarized in Table 2). In some human and mouse mammary tumors, ROS levels in CSCs are lower than those found in corresponding nontumorigenic cells (NTCs). Compared to NTCs, CSCs show less DNA damage and are more viable. A highly activated free radical scavenger system contributes to lower levels of ROS in CSCs. Pharmacologic depletion of ROS scavengers in CSCs significantly decreases their ability to form colonies, leading to increased radiosensitivity [51]. These suggest that, similar to stem cells, CSCs in tumors can enhance reactive oxygen defense and reduce ROS levels, which may lead to cancer radiotherapy resistance [15]. Recent reports confirm that ROS are associated with GC stem cell markers CD133, CD166, and CD44 [52–54]; ROS can also regulate EMT-related indicators, such as E-cadherin, N-cadherin, snail, and twist [55].

**Figure 3:**
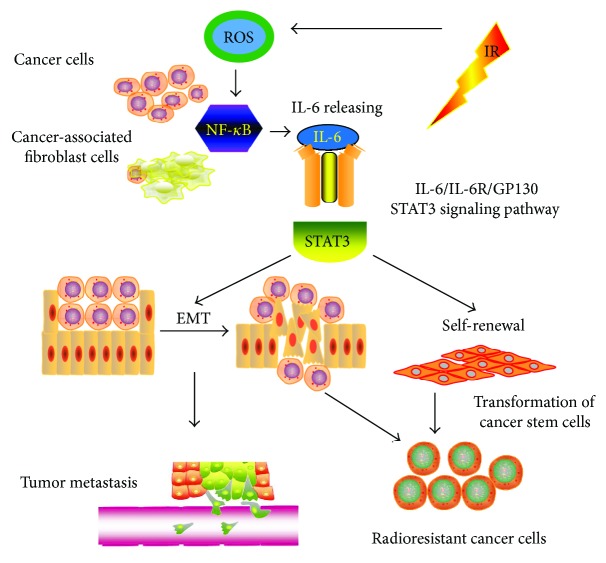
The IL-6R/gp130/STAT3 signaling pathway. The IL-6 secreted by tumor cells and CAFs through the ROS-mediated NF-*κ*B signaling pathway can promote tumor metastasis, radioresistance, and CSC self-renewal. IL-6 promotes GC metastasis and CSC self-renewal through the classical IL-6R/gp130/STAT3 signaling pathway.

EMT is crucial not only in regulating tissue development but also in tumor invasion and metastasis [56]. The change of the microenvironment plays an important role in the development of tumors, stem cell transfer, and self-renewal. ROS change the tumor microenvironment by regulating a variety of cell signaling pathways to promote CSC transformation [57, 58]. ROS also regulate the activity of NF-*κ*B, which is an important mediator of the release of inflammatory factors by tumor cells [59, 60]. In breast cancer, head and neck squamous cell carcinoma, gastric cancer, and glioma, IL-6 promotes stem cell self-renewal through the classical IL-6R/gp130/STAT3 signaling pathway [61] (summarized in Figure 3). An elevated level of IL-6 is related to cancer cell proliferation, angiogenesis, and metastasis via stimulation of MAPK, STAT3, and AKT signaling pathways [62, 63]. IL-6 accelerates EMT through an altered expression of N-cadherin, E-cadherin, twist, snail, and vimentin, which results in cancer metastasis. It is reported that the levels of ROS, IL-6, COX2, and TNF-*α* are abnormally increased in patients with GC. Therefore, ROS may activate NF-*κ*B to cause GC cells and cancer-associated fibroblast cells (CAFs) to release IL-6, thus mediating tumor metastasis and self-renewal that will consequently facilitate CSC self-renewal and maintenance. The activation of the cellular DNA damage checkpoint and the ability of DNA repair in CSCs result in their survival of radiotherapy, thus establishing radioresistance in GC cells.

### 1.4. Targeting ROS and ROS-Associated Tumor Microenvironment Signaling Pathways

Recently, multiple medicinal and chemical therapies are investigated to target the factors and signaling pathways associated with ROS-mediated TME alteration, ROS-mediated DNA damage, and apoptosis (summarized in Table 3). Selenium nanoparticles (SeNPs) possess special chemical and physical properties and generate ROS in cells to provide a novel strategy for the rational design and synthesis of chemo-radiosensitizing therapeutic materials [65]; SeNPs are also confirmed to affect on TNF and IRF1 to induce ROS-mediated activation of necroptosis [70]. There are also reports demonstrating that an increased level of ROS is a feasible strategy to improve radiotherapy efficacy [64–66]. Most of the drugs like bortezomib, celecoxib, 5-FU, and other compounds are validated to enhance the generation of ROS. Other reports suggest that microRNAs and other materials can repress the factors in ROS-mediated TME and enhance radiosensitivity in numerous cancer cells, including GC cells [72, 73]. Nonetheless, the mechanisms of ROS-mediated TME alteration in GC are not explicitly understood, particularly regarding key genes and proteins that influence the signaling pathways within the TME, inflammatory factors releasing, CSCs, EMT, and ROS scavenging.

## 2. Conclusion

The purpose of radiotherapy is to eliminate tumor cells, but spare normal cells and tissues from radiotherapy damage. However, currently single-course radiotherapy cannot provide sufficiently high-dosage radiotherapy for effective treatment of GC. While ROS can be induced chronically during multiple rounds of radiotherapy, its antitumor effect may be compromised. Within the tumor microenvironment, radiotherapy and several key cytokines can promote ROS production, consequently suppressing the antioxidant system. Patients with GC suffer from chronic oxidative stress and have higher levels of locally induced ROS, which leads to an abnormal expression of cytokines and inflammatory factors. ROS can activate a variety of signal molecules, such as MAPK, NF-*κ*B, TNF-*α*, and TGF-*β* that transform the TME by releasing inflammatory factors, including IL-1*β*, IL-6, COX-2, TNF, and NOX2. These inflammatory factors promote the development and progression of GC through cellular proliferation and apoptosis signal pathways. The GC stem cell markers like CD133, CD166, and CD44 are also associated with ROS and EMT markers. ROS can also activate several cell signal pathways to regulate the CSCs. ROS-activated NF-*κ*B mediates the release of IL-6 in GC, breast cancer, glioma, and HNSCC. The IL6R/gp130/STAT3 signal pathway regulates CSC renewal and cancer metastasis which leads to radiotherapy resistance. Further investigation of treatment options addressing the pathways associated with ROS in GC may increase the sensitivity of radiotherapy in patients with GC. Since inflammatory factors play an important role in ROS-mediated TME alteration and CSCs, anti-inflammatory drugs, such as NSAIDs and glucocorticoids, can be used to regulate the release of inflammatory factors and restore the aberrant TME. In addition, an optimal dosage of radiotherapy in less therapeutic frames of radiotherapy, along with other strategies to increase radiosensitivity, may significantly augment effective ROS levels for GC treatment.

## Figures and Tables

**Table 1:** ROS and TME-relevant signaling.

ROS target	Factors	Signaling pathways	Function	References
P38	TLR2/6	P38 MAPK, JNK	Translocation of NF-*κ*B to the nucleus	[26]
NF-*κ*B	IL-1*β*, IL-6, COX-2, IL-2, IL-8, TNF, NOX2, CXCL12	STAT3, NF-*κ*B p65	Regulation of tumor proliferation and apoptosis	[23, 27]
TNF	TNFs	TNF, NF-*κ*B, JNK	Cell survival or death	[27]
TGF-*β*		TGF	EMT inducer	[28, 30]
NOX-3	NADPH	MAPK, STAT1	Inflammation and apoptosis	[31]

**Table 2:** CSCs involve in the mechanisms of radioresistance.

Mechanism	Signaling pathways	References
Protection of DNA repair	PARP ATR-Chk1 ATR-Cnk1, ATM-Chk2 Chk1, Chk2 ATM-ZEB1-Chk1 Myc-Chk1 and Chk2 AKT/cyclin D1/Cdk4 Upregulated DNA repair genes	[35] [36] [37] [38] [39] [40] [41] [42, 43]
Protection of ROS scavenging	Nrf2 signaling pathway The Prdx family of antioxidant enzymes	[44–46] [47, 48]
Protection of TME change	HIF-mediated mechanisms and negative immune responses	[14, 18, 49, 50]

**Table 3:** Novel therapies targeting the ROS-mediated TME alteration.

Therapy	Target	Material type	Mechanism	References
BEMER electromagnetic field therapy	ROS	Cancer cell lines	Enhanced ROS formation and induced DNA damage	[64]
X-ray responsive selenium nanoparticles	ROS	HeLa and NIH3T3 cells	ROS overproduction causing the cell apoptosis	[65]
Diisopropylamine dichloroacetate	ROS	Human esophageal squamous cell carcinoma cell lines Eca-109 and TE-13	Modulated mitochondrial oxidation	[66]
Bortezomib, romidepsin	NF-*κ*B	Human NSCLC cell lines (A549)	Increasing ROS and stimulating the extrinsic pathway of apoptosis	[67]
Bortezomib	ROS, Noxa	Mantle-cell lymphoma cell lines and patients	Cytotoxic effect through ROS generation and Noxa induction	[68]
Celecoxib, 5-FU	ROS	Human squamous cell lines (SNU-1041 and SNU-1076), orthotopic tongue cancer mouse model	Inhibiting the AKT pathway and enhancing ROS production	[69]
Selenium nanoparticles	TNF, IRF1	Human prostate adenocarcinoma cell line (PC-3)	Causing TNF and IRF1-induced ROS-mediated necroptosis	[70]
miR-139-5p	Multiple genes	Breast cancer patients, human breast cancer cell line (MCF7), xenograft mouse model	Suppression of gene networks of DNA repair and ROS defense	[71]
Ursolic acid		BGC-823 human adenocarcinoma gastric cancer cell line	Enhanced G2/M arrest, increasing ROS, promoting apoptosis	[72]
miR-200c nanoparticles	CSC	Human gastric adenocarcinoma cell lines (BGC823, SGC7901, and MKN45) and an immortalized human gastric mucosa cell line (GES-1)	Impairing ROS generation and DNA repair by the miR-200c	[73]

## Additional Points

*Highlights*. (i) This review focuses on recent advances in the research of radiotherapy-mediated tumor microenvironment changes in gastric cancer. (ii) There is accumulating evidence that reactive oxygen species induce the transformation of the tumor microenvironment that promotes cancer stem cells and epithelial-mesenchymal transition. (iii) This review proposes key targets for improving the radiosensitivity of gastric cancer.
